# Top-down norms and psychological empowerment: Explaining Chinese public acceptance of autonomous driving

**DOI:** 10.1371/journal.pone.0331911

**Published:** 2025-09-11

**Authors:** Wenjun Liao, Xukang Liu, Kaixuan Jiang, Xiangqun Liu, Jianjun Yang, Jia Chen

**Affiliations:** 1 School of Automobile and Transportation, Xihua University, Chengdu, China; 2 Xihua Jiaotong Forensics Center, Chengdu, China; 3 Academy of Intelligent Manufacturing and Vehicle Engineering, Chengdu Vocational and Technical College of Industry, Chengdu, China; Shandong University, CHINA

## Abstract

This study explains Chinese public acceptance of autonomous driving (n = 412) through subjective norms, personal norms, and psychological empowerment (cognitive, emotional, behavioral). Using covariance-based SEM with bootstrapped mediation, we find behavioral empowerment has the strongest direct effect on acceptance (≈2 × cognitive; ≈ 3.2 × emotional). All three empowerment dimensions partially mediate both subjective- and personal-norm paths, with behavioral mediation (≈46–50%) dominating. Importance–Performance Analysis highlights behavioral empowerment as high-importance/high-performance, while cognitive empowerment shows headroom for improvement. A Bayesian network confirms predictive validity with low overall error. Findings suggest design and policy should primarily strengthen users’ behavioral empowerment (capability, controllability, tangible benefits), while complementing cognitive and emotional pathways. We also discuss China-specific “top-down” cultural mechanisms that amplify subjective norms and outline implications for cross-cultural generalization.

## 1. Introduction

### 1.1. Background and significance of the study

Autonomous driving (driverless) uses computer science and AI to run vehicles fully, safely, and efficiently without human input. Advances in AI, mobile internet, and big data make it a core part of intelligent transportation systems and a practical fix for current transport problems [1]. Governments worldwide are increasing R&D, pilot testing, and deployment, while issuing supportive rules and standards. Boston Consulting Group projects sustained growth from 2018, with autonomous vehicles reaching 25% of global new-car sales by 2035 and US$77 billion in industry value [2]. China, the world’s largest auto market, is shaped by culture, policy, and market forces. Together with rapid technical progress, these factors are pushing China toward an era of intelligent autonomous mobility.

On the one hand, the widespread adoption of autonomous driving is contingent upon advancements in scientific and technological capabilities. On the other hand, the public’s acceptance of this technology is indicative of the societal demand for autonomous driving technology [3–5]. As autonomous driving technology becomes embedded in everyday life, scholarship on public acceptance has expanded accordingly. Given humans’ inherently social nature, social norms have emerged as a critical determinant of acceptance [6–8]. Social norms, in this context, encompass both subjective norms and personal norms, which derive from the Theory of Planned Behavior and the Norm Activation Theory, respectively. “Subjective norms” refer to an individual’s perceptions of specific behaviors, shaped by the influence of significant others or societal groups such as family, friends, customs, moral standards, legal frameworks, and media [9]. Through the evaluation and viewpoints of these influences, individuals form value judgments regarding particular actions. In contrast, “personal norms” relate to an individual’s internal expectations to perform a specific behavior within a given context. These norms represent the internalization of social norms and reflect an individual’s moral sense of responsibility. Violating personal norms induces feelings of guilt, self-denial, or a loss of self-esteem, while adherence to these norms can foster pride and enhance self-worth [10]. Subjective norms capture the influence of others’ evaluations on behavioral intentions, whereas personal norms reflect the internalization and enactment of moral obligations at the individual level. Social norms are therefore central to public acceptance of autonomous driving. However, prior research has concentrated on subjective norms and often neglected personal norms, especially individuals’ moral perspectives. In collectivist contexts such as China, subjective norms, including conformity and perceived social expectations, exert stronger effects on technology adoption than in individualistic cultures. Finally, despite increasingly comprehensive models, the role of psychological empowerment in shaping public acceptance remains underexplored.

### 1.2. Literature review

Previous research has predominantly employed two models to assess acceptance: the Technology Acceptance Model (TAM) [11] and the Unified Theory of Acceptance and Use of Technology (UTAUT) [12]. TAM identifies perceived usefulness and perceived ease of use as core drivers of adoption. Given the distinct features of autonomous driving, researchers have extended TAM in two main ways. First, they added antecedent variables such as environmental concern, perceived risk, and trust to explain public acceptance [13–15]. For example, Wu et al. [13] separated perceived risk into personal-safety and privacy components and showed that environmental concern and trust promote acceptance, whereas perceived risk reduces it. Second, they refined outcome measures by disaggregating acceptance into attitudes, willingness to pay, and usage preferences. Evidence indicates that trust, perceived benefits, and perceived risks are strongly associated with these dimensions [14]. The UTAUT model includes four core dimensions: performance expectancy, effort expectancy, social influence, and facilitating conditions. Extensions of UTAUT have incorporated additional factors. Leicht et al. [16] examined consumer innovativeness by classifying it as high or low and reported that performance expectancy, effort expectancy, and social influence were positively associated with willingness to pay, with innovativeness strengthening these effects. Hewitt et al. [17] added attitude, self-efficacy, anxiety, and perceived safety and confirmed significant effects on intention to use, while also observing that acceptance declined as automation levels increased. Ro et al. [18] introduced convenience, safety, cost, economic use, legal liability, and decision rules, showing that these variables directly affected attitude, which in turn influenced acceptance. Building on TAM and UTAUT, Nordhoff et al. [19] proposed the Autonomous Vehicles Acceptance Model (AVAM), which integrates sociodemographics, travel factors, vehicle characteristics, environmental variables, and psychological and emotional aspects. Beyond attitudinal models, discrete choice studies further clarify determinants of acceptance. Jiang et al. [20] used a mixed multinomial logit model and found that respondents were willing to pay for certain autonomous features; parking costs, insurance premiums, market share, and time to market significantly affected willingness to pay. Cyganski et al. [21] employed a multinomial probit model and reported higher future use among male respondents, frequent travelers, and those with more work-related travel. Qualitative research also contributes. Harrow et al. [22] combined workshops with questionnaires, drawings, physical models, videos, and commentaries to elicit preferences for autonomous driving.

Methodologically, many studies relied on descriptive analyses that are insufficient to uncover nuanced patterns. TAM and its extensions provide limited leverage for estimating price elasticities and marginal effects in the autonomous-driving context. Econometric approaches such as multinomial logit and structural equation models rest on strong assumptions. It is therefore essential to test whether autonomous-driving travel-behavior data satisfy these assumptions and to adjust model specifications when they do not.

Based on previous research, the key factors influencing the public acceptance of autonomous driving technology can generally be categorized into six areas: vehicle safety, automation level, travel-related attributes, environmental factors, individual characteristics, and social norms. It is important to note that “individual characteristics” here differs from the concept of “personal norms” introduced in this study. The former refers to personal attributes such as gender, age, occupation, income, residence, education level, family structure, and driving experience. Regarding vehicle safety, Xu et al. [23] reported a significant positive association between safety levels and acceptance. Dong [24] found that vehicles with in-car human supervision were perceived as safer and were more acceptable. Privacy concerns are salient, particularly in developed countries, where individuals prefer vehicles that do not require data sharing [25]. The level of automation and related attributes also matter. Many studies found that acceptance generally declined as automation increased [25–28], although Bansal et al. [29,30] reported higher willingness to pay for fully autonomous vehicles than for partially automated ones. Sivak et al. [31] noted that additional functions can increase in-vehicle activities but may also induce severe motion sickness, which lowers acceptance. Price is another key determinant. Shabanpour et al. [32] reported high sensitivity to purchase cost, and Bansal et al. [33] projected that a 5% annual price decrease for fully autonomous vehicles would lead to a 24.8% market share on U.S. roads by 2045. Travel-related attributes include cost, time, distance, and experience. Several studies found that lower cost differentials between autonomous and conventional options increase acceptance [18,34–36]. Individuals generally expect autonomous driving to reduce travel time and are willing to pay a premium for this benefit [1]. Travel distance showed no significant effect in Bansal et al. [29]. Prior experience also shapes preferences: frequent first-class train passengers were more likely to choose autonomous vehicles for last-mile travel [34], and users with broader experience of shared products were more willing to adopt shared autonomous vehicles [35]. Environmental factors encompass use models and deployment scenarios. These contexts influence consumers through cognitive and affective appraisals, with Sofi et al. [37] conceptualizing and validating added constructs that illuminate how environmental conditions channel adoption decisions. Nielsen et al. [38] found a stronger inclination toward private ownership than shared use, whereas Webb et al. [39,40] showed that understanding the benefits increased willingness to use shared autonomous vehicles. Built environment and accessibility also influence adoption [41]. Payre et al. [29,42] reported greater willingness to use autonomous vehicles in high-speed or congested settings to reduce fatigue. Perceptions of local traffic environments shape attitudes toward autonomous vehicles [43]. In a survey of 421 French car users, Payre et al. [42] found that approximately 71% were willing to adopt fully autonomous vehicles, and 45% of respondents frequently consumed alcohol, suggesting potential appeal when driving is inadvisable. Findings on individual characteristics are mixed. Many studies reported that men were more interested and showed higher willingness to pay or use [29,42,44,45], while others found greater willingness among women [26,27,46,47] or no gender differences [28]. Evidence on age is also inconsistent: some studies found higher willingness to pay and intention to use among younger respondents [27,29,35,48–50], whereas Rǒedel et al. [26] observed stronger intention among older respondents, and Payre et al. [42] reported no age effect. Income was positively associated with willingness to pay and use in several studies [27,44], although Sivak et al. [31] found no significant effect. Residence matters: urban respondents expressed higher anticipation than suburban respondents [27,51–53]. Family structure and marital status also play roles. Households with children were more open to autonomous driving [44], and married respondents were generally more receptive, although parents with children who enjoy driving were less willing [39]. Education and ownership show divergent trends. Higher education and car-free households were associated with more favorable attitudes [54], yet car owners reported greater eagerness, willingness to purchase, and willingness to use [27]. Greater driving experience also correlated with higher acceptance [44]. Schoettle et al. [27] reported higher preference for autonomous taxis in lower-income countries. Respondents from China and India expressed more positive attitudes than those from Japan, the United States, the United Kingdom, and Australia, although safety concerns about full automation were common across all groups, consistent with Anania et al. [55]. Social norms include social relationships, legal and regulatory conditions, institutional influences, ethics, and the moral responsibilities formed under these influences. When family and peers use autonomous vehicles or express positive views, individual willingness to adopt increases [56]. Clear liability frameworks are widely regarded as essential for public acceptance and for integrating highly automated vehicles on public roads [18,24,57–59]. Insurance coverage for autonomous-vehicle crashes raises acceptance [27]. In discrete choice experiments, relieving drivers of liability and providing dedicated lanes increased acceptance [32]. Evidence from adjacent domains indicates that normative cues and consumption emotions guide real-world choices, with descriptive norms and consumption emotions significantly influencing satisfaction judgments [60,61].Ambiguous liability, weak enforcement against illegal driving, and unregulated driving ethics reduce willingness to adopt [62,63].

It is important to distinguish subjective norms from personal norms. Prior research mainly operationalized social norms as subjective norms that reflect perceived expectations of others, which encourages a bottom-up acceptance process starting from individual users. Personal norms center on moral responsibility and are shaped by cultural context [10,64–66]. In collectivist societies such as China, conformity, social expectations, and respect for authority are prominent. Hofstede’s cultural dimensions suggest a top-down acceptance mechanism, whereby family, society, and government policies strongly influence public attitudes [67,68]. Similar patterns have been observed in studies of emotional AI. In collectivist cultures, government and social authority exert stronger influence, which can raise acceptance of new technologies [69,70]. Compared with Western cultures that emphasize individual choice, the Chinese public is more likely to accept new technologies under the guidance of social and institutional structures. This tendency often appears as a top-down cognitive and behavioral adjustment driven by government, media, and collective society [71–73].

Psychological empowerment offers a complementary lens. Zimmerman defined empowerment as a process through which external interventions enhance individuals’ capabilities and awareness of rights, reduce powerlessness, and promote social change [74]. Psychological empowerment focuses on internal experiences and perceptions [75]. The concept has been widely applied in feminism, tourism, education, and research on marginalized groups [76–79], and it has begun to inform studies of public acceptance of autonomous driving. Sara H. Hsieh et al. [80] identified perceived responsiveness and care as social factors that foster psychological empowerment. Divine Q. Agozie et al. [81] found a positive association between empowerment and attitude and observed an indirect effect on purchase intention. Julien François et al. [82] showed that psychological health applications improved empowerment among individuals with mental health disorders and increased trust in providers. These findings indicate that examining public acceptance of autonomous driving through the lens of psychological empowerment is a valuable direction for future research.

In summary, current research on the public acceptance of autonomous driving technology faces two key limitations. First, studies on social norms often neglect the role of personal norms. Second, the influence of psychological empowerment on user acceptance has been largely overlooked. Therefore, this study incorporates personal norms, drawn from the theory of norm activation, as an influencing factor and investigates its impact alongside subjective norms on the public acceptance of autonomous driving technology. This enriches the understanding of the determinants of acceptance. Additionally, from the lens of psychological empowerment, this research examines the “perception-behavior” paradigm and its influence on public acceptance. By applying psychological empowerment theory within the context of autonomous driving, a novel research model is introduced, laying a theoretical foundation for future research in the domain of intelligent driving.

## 2. Materials and method

### 2.1. Choice of method

We used covariance-based structural equation modeling in AMOS because the study was confirmatory, the constructs were reflective and multi-item, and our goal was to recover the covariance structure, assess global fit, and quantify direct and indirect effects. We reviewed alternatives and found them misaligned with these aims: PLS-SEM is mainly prediction-oriented and lacks CB-style global fit; logistic or ordered models cannot represent the latent measurement model and limit mediation testing; multilevel modeling requires a verified hierarchical sample that our data do not provide; MIMIC or IRT emphasize item calibration rather than relations among multiple constructs.

CB-SEM with maximum likelihood (ML) assumes approximate multivariate normality. We examined univariate skewness/kurtosis for all indicators and multivariate normality using Mardia’s kurtosis in AMOS. Item-level skewness ranged from −0.904 (SN3) to +0.696 (SN4) and kurtosis from −0.572 (CE3) to +1.559 (CE2). At the multivariate level, Mardia’s kurtosis = 19.979; CR = 6.835, indicating mild multivariate non-normality. Accordingly, we estimated the model with ML and implemented a 5,000-sample Bollen–Stine bootstrap to obtain a robust χ² p-value and bias-corrected (BCa) 95% confidence intervals for structural paths and indirect effects. To strengthen credibility and practical usefulness, we also complemented CB-SEM with a bootstrap mediation check to corroborate indirect effects, an Importance–Performance Analysis to translate effects and performance into priorities, and a Bayesian network to examine out-of-sample predictive validity under potential distributional shifts, which fit indices alone do not guarantee.

### 2.2. Models and hypotheses

#### 2.2.1. The impact of psychological empowerment on the acceptance of autonomous driving technology.

Empowerment involves granting power, encouraging users to actively engage in decision-making and activities. Christens (2012) [83] categorized psychological empowerment into three dimensions: cognitive empowerment—skills and critical understanding for exerting social influence; emotional empowerment—self-perception of one’s ability to influence socially; and behavioral empowerment—the direct impact of actions taken. In autonomous driving, empowerment captures the extent to which the technology helps users perform better or reduces effort. Consistent with this view, Li et al. [84] found that empowerment strengthens feelings of usefulness, enjoyment, empathy, and trust, reinforcing emotional and normative commitment and thereby encouraging supportive behavior. Huang (2023) [85] showed that perceived usefulness and ease of use raise attitudes, usefulness directly increases intention, and ease of use strongly enhances usefulness; enjoyment and trust also emerge as positive predictors of intention—together substantiating that cognitive empowerment (clarity/usefulness) and emotional empowerment (enjoyment/trust) increase acceptance. Complementing this, Zefreh et al. [86] found that effort expectancy elevates performance expectancy and, via performance expectancy, intention—evidence that behavioral empowerment promotes acceptance. Based on this, the following hypotheses are proposed:

H1: Cognitive empowerment positively influences the public acceptance of autonomous driving technology.
H2: Emotional empowerment positively influences the public acceptance of autonomous driving technology.
H3: Behavioral empowerment positively influences the public acceptance of autonomous driving technology.

#### 2.2.2. The impact of personal norms on users’ psychological empowerment.

Personal norms, as introduced by Schwartz in the Norm Activation Theory (1977) [10], help elucidate the mechanisms through which personal norms are activated into prosocial behaviors. This theory highlights the moral obligation to engage in pro-environmental actions. Extending TPB and VBN frameworks, Wu et al. [87] showed that personal norms directly predicted behavior and were intertwined with cognitive constructs such as environmental knowledge and concern, while perceived behavioral control, a proxy for capability and required effort, operated alongside norms on the path to behavior. Rosenthal et al. [88] likewise demonstrated that anticipated guilt, an affective mechanism closely tied to personal norms, reliably motivated civic anti-littering engagement, which highlighted a personal-norms–emotion–behavior sequence. In an integrated NAM and TPB model, Savari et al. [89] confirmed a cognitive chain from awareness of consequences and assignment of responsibility to personal norms and then to intention, and identified perceived behavioral control and subjective norms as among the strongest predictors of pro-environmental intention. Autonomous driving technology is considered a sustainable transportation option and a key strategy for mitigating urban traffic congestion [90,91], making it a form of pro-environmental behavior. Therefore, applying the concept of personal norms to examine their impact on the public acceptance of autonomous driving technology is highly appropriate. Accordingly, we posited that stronger personal norms would raise cognitive empowerment through greater clarity and understanding, strengthen emotional empowerment through pro-social affect such as anticipated guilt, and enhance behavioral empowerment through higher capability with lower effort, thereby increasing acceptance. The hypotheses are presented as follows:

H4: Personal norms positively influence cognitive empowerment.
H5: Personal norms positively influence emotional empowerment.
H6: Personal norms positively influence behavioral empowerment.

#### 2.2.3. The impact of subjective norms on users’ psychological empowerment and personal norms.

Subjective norms, a key component of the Theory of Planned Behavior (TPB), have been increasingly applied in the automotive field. Liu et al. [92] examined young people’s intention to use shared autonomous vehicles within an extended TPB–TAM model and found that subjective norms significantly increased perceived usefulness, which in turn enhanced initial trust and attitude, thereby indirectly improving behavioral intention. This evidence supported both cognitive empowerment through usefulness and emotional empowerment through trust. In addition, Wu et al. [93] demonstrated that electronic word-of-mouth and mass media strengthened adoption intention by enhancing trust and self-efficacy through subjective norms. These findings provided direct support for emotional empowerment via trust and behavioral empowerment via self-efficacy and highlighted the pivotal role of subjective norms in empowerment pathways. Furthermore, researchers have integrated TPB with Norm Activation Theory in the health and environmental domains, confirming that subjective norms influence personal norms [81]. Based on this, the present study incorporates subjective norms into its framework, leading to the following hypotheses:

H7: Subjective norms positively influence cognitive empowerment.
H8: Subjective norms positively influence emotional empowerment.
H9: Subjective norms positively influence behavioral empowerment.
H10: Subjective norms positively influence personal norms.

**Fig 1:**
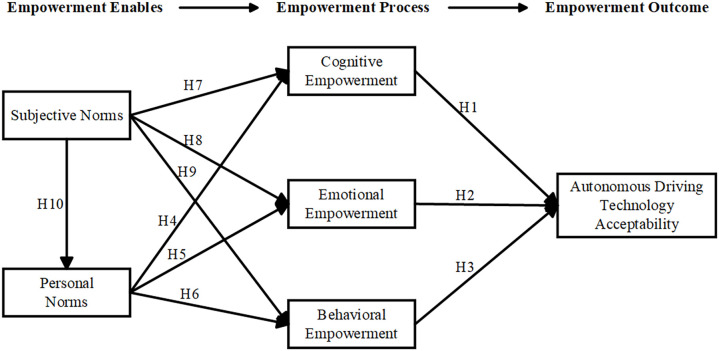
The autonomous driving acceptance model based on users’ psychological empowerment.

Building on the aforementioned hypotheses, this study develops a model to explore the public acceptance of autonomous driving technology. The model positions subjective norms and personal norms as the foundational factors, with the three dimensions of psychological empowerment acting as mediators and direct factors. The proposed research framework is presented in Fig 1.

### 2.3. Data collection

#### 2.3.1. Questionnaire.

This study employed a literature review methodology to conduct a comparative analysis of existing questionnaires. Concurrently, five experts in the field of intelligent vehicles were engaged to evaluate the questionnaire we developed. Ultimately, to ensure both content validity and participant psychological considerations, the questionnaire was structured into three distinct sections. The initial section consisted of textual introductions to autonomous driving vehicles and autonomous driving technology. The second section gathered participant demographic information, including gender, age, education level, marital and parental status, annual income, and daily commuting distance. The third section comprised the specific observation items, employing a five-point Likert scale, with a total of six latent variables and twenty observed variables. The detailed contents of the scale are presented in Table 1.

**Table 1:** Autonomous driving technology acceptance survey scale.

Variants	Measurement items	Source
Subjective Norms (SN)	SN1: People around me (family, friends, etc.) will support my choice of an autonomous vehicle	Nielsen and Haustein (2018) Hajjafari (2018)
	SN2: The publicity from the companies, the guidance from the government will make me think more about autonomous vehicles.	
	SN3: Legal and moral support for autonomous vehicles would also motivate me to choose autonomous driving cars	
	SN4: I think autonomous vehicles have a great future.	
Personal Norms (PN)	PN1: I’m open to trying and using autonomous vehicles	Schwartz (1977)
	PN2: Proactively embracing new technology (autonomous driving technology) is consistent with my demonstration of social responsibility	
	PN3: I believe I have a responsibility and an obligation to support the advancement of new technologies, including autonomous vehicles.	
	PN4: Autonomous driving technology improves my quality of life, which is in line with my personal standard of living.	
Cognitive Empowerment (CE)	CE1: Autonomous driving technology has given me a better understanding of where the automotive industry is headed	Christens (2012)
	CE2: Autonomous driving technology guides my driving maneuvers	
	CE3: Autonomous driving technology has made me more aware of my travel planning	
Emotional Empowerment (EE)	EE1: Using autonomous vehicles can make my life more enjoyable	Speer and Peterson (2000)
	EE2: With autonomous vehicles, I’ll be able to enjoy traveling even more.	
	EE3: Using an autonomous vehicle makes me feel confident	
Behavioral Empowerment (BE)	BE1: Autonomous vehicles facilitate our personal mobility	Speer and Peterson (2000)
	BE2: The development of autonomous vehicles will improve my quality of life	
	BE3: Autonomous driving technology enhances our driving experience	
Autonomous Driving Technology Acceptance (AC)	AC1: I would be willing to try to ride or drive an autonomous driving car.	Nielsen and Haustein (2018) Hajjafari (2018)
	AC2: I would encourage family and friends to try out autonomous vehicles	
	AC3: I would like to contribute to the development of autonomous driving technology	

#### 2.3.2. Procedure.

The questionnaire was released on “Wenjuan.com” (www.wenjuan.com) on June 10, 2024, and closed on June 30, 2024, with a total of 447 responses collected. After data cleaning, questionnaires with completion times under 200 seconds and those with identical responses across all items were excluded. As a result, 412 valid questionnaires were retained, yielding a response rate of 92.17%.

Notably, although online surveys may introduce self-selection bias, they are widely used in large-scale studies and can reach diverse social groups. In China, where internet penetration exceeds 90%, the broad use of platforms such as “Wenjuan.com” facilitates coverage across regions, ages, and education levels. Accordingly, it is reasonable to use this platform to collect sample data [94].

Self-selection is inherent in online surveys, especially in technology-acceptance research on innovative products that naturally attract more technology-positive respondents. This pattern can also indicate public interest and provide baseline data for market trends. To enhance diversity and limit the influence of any single bias, this study used stratified and random sampling across geographic areas, social backgrounds, age groups, and education levels.

Although some participants may lack direct experience with autonomous driving, this does not unduly compromise their assessments. Public acceptance often reflects perceived attributes of the technology, expectations about future applications, and external discourse, including media reports. Prior work shows that perceptions and social expectations can outweigh firsthand experience [95].

#### 2.3.3. Participants.

Statistical inference is limited to adult Chinese internet users with high exposure to autonomous-driving information and services. Because online recruitment produced an overrepresentation of higher-income and higher-educated respondents and many with prior exposure, the realized sample reflects an early-exposure or early-adopter segment rather than a nationally representative cross-section. Demographic profiles are reported in Table 2 for transparency, not as evidence of national representativeness. This segment remains analytically salient for near-term design and policy: early-exposure groups disproportionately shape expectations, usage norms, and diffusion, and attitudes toward autonomous driving are formed through information, observation, and public discourse as well as direct use. Accordingly, all substantive claims are bounded to this segment, and limited representativeness is acknowledged as a study constraint. Potential heterogeneity across city tiers and local exposure environments is also recognized and merits examination in subsequent work through stratified sampling and moderation or multilevel analyses as data permit.

**Table 2:** Statistical table of basic information of participants.

Variables	Classifications	Counts	Percentages
Gender	Male	239	58.01%
	Female	173	41.99%
Age	18–25 years old	127	30.83%
	26–35 years old	180	43.69%
	35–45 years old	86	20.87%
	>45 years old	19	4.61%
Education	Junior high school and below	23	5.58%
	Senior high school	91	22.09%
	College	246	59.71%
	Master’s degree and above	52	12.62%
Marriage and childbearing	Unmarried	153	37.14%
	Married but not having children	43	10.44%
	Married and having children	216	52.43%
Annual income	<100,000	69	16.75%
	100,000–200,000	189	45.87%
	200,000–300,000	105	25.49%
	300,000–500,000	37	8.98%
	>500,000	12	2.91%
Daily commuting distance	<5km	11	2.67%
	5km–10 km	103	25.00%
	10km–15 km	167	40.53%
	15km–20 km	68	16.50%
	20km–30 km	44	10.68%
	>30km	19	4.61%
Driving experience	None	43	10.44%
	0–2 years	55	13.35%
	2–5 years	116	28.16%
	5–10 years	179	43.45%
	over 10 years	19	4.61%
Experience with autonomous driving technology	No	71	17.23%
	Yes	341	82.77%

### 2.4. Statement

(1) This study was approved by the Ethics Committee of the School of Automotive and Transportation, Xihua University (Approval No. 2024LL(01)) and was conducted in accordance with the Declaration of Helsinki. Informed consent was obtained from all subjects for this study. We obtained written consent from the participants, and this evidence can be downloaded from the Wenjuan.com platform.
(2) The survey started on June 10, 2024, and ended on June 30, 2024. At the beginning of the questionnaire, we briefly presented our team’s background, the experiment details, and the purpose of the survey results, while ensuring participants’ privacy protection. After this, we posted the following question on “Wenjuan.com”: ‘Have you been fully informed and agreed to participate in this survey? (If you are a minor, please ensure your guardian is informed and agrees).’ We set the system so that only participants who selected ‘Yes’ could proceed with the questionnaire, while those who selected ‘No’ were considered to have declined and could not continue.
(3) The results of this study were obtained with the participants’ informed consent, and throughout and after the survey period, the information collected was limited to what was specified in the questionnaire. Additionally, no other personal information of the participants was collected. Therefore, the survey findings are suitable for long-term use by researchers.

## 3. Data analysis and results

### 3.1. Common method bias

Since the dataset of this study was obtained through cross-sectional and self-reported surveys, a common method bias test was conducted for this study. The results of the Harman’s single-factor test showed that the explained rate of the first factor was 38.1%, which was lower than the critical criterion of 40%, indicating that the data of the present study did not suffer from serious common method bias phenomenon.

### 3.2. Confirmatory factor analysis

This study employed Structural Equation Modeling (SEM) to test the measurement items and the research model, utilizing SPSS and AMOS software for data analysis. First, the goodness of fit of the model was assessed, with CMIN/DF, RMSEA, GFI, IFI, TLI, and CFI selected as fit indices. As shown in Table 3, the structural equation model demonstrated a high degree of fit with the sample data, indicating that the model is suitable. Additionally, univariate diagnostics showed modest departures from normality (skewness −0.904 to +0.696; kurtosis −0.572 to +1.559), and Mardia’s kurtosis was 19.979 (CR = 6.835), evidencing mild multivariate non-normality. We therefore report the Bollen–Stine bootstrap χ² p-value = .019 (5,000 resamples) together with BCa 95% CIs for paths and indirect effects; all substantive conclusions remained unchanged relative to conventional ML inference.

**Table 3:** Parameter values of model fit goodness-of-fit indicators.

Norm	Reference standard	Actual results
CMIN/DF	1-3 as excellent, 3–5 as good	1.371
RMSEA	<0.05 as excellent, < 0.08 as good	0.030
GFI	>0.9 as excellent, > 0.8 as good	0.950
IFI	>0.9 as excellent, > 0.8 as good	0.984
TLI	>0.9 as excellent, > 0.8 as good	0.981
CFI	>0.9 as excellent, > 0.8 as good	0.984

Internal consistency was assessed using Cronbach’s Alpha (α) and Composite Reliability (CR). A model is considered to have good internal consistency if α > 0.7 and CR > 0.7. Factor Loadings (FL) and Average Variance Extracted (AVE) were used to assess convergent and discriminant validity. A measurement model is considered to have good convergent validity if FL > 0.6 and AVE > 0.5. Discriminant validity is confirmed if the square root of AVE for each variable is greater than the correlation coefficients between that variable and others. As shown in Table 4, all latent variables have α values greater than 0.7, indicating good internal consistency of the sample data. All factor loadings exceed 0.60 and all critical ratios are large (|CR| > 3.29, p < 0.001), indicating statistically significant loadings. The CR values for all latent variables are greater than 0.7, and the AVE values exceed 0.5, demonstrating strong explanatory power and good convergence. Table 5 shows that the square roots of the AVEs for all variables are greater than the correlation coefficients with other variables, confirming good discriminant validity for the model.

**Table 4:** Results of the confirmatory factor analysis.

Variables	Measurement items	Critical Ratio	FL	Cronbach’s Alpha	CR	AVE
Subjective Norms (SN)	SN1		0.755	0.837	0.835	0.560
	SN2	13.996***	0.715			
	SN3	15.922***	0.814			
	SN4	13.744***	0.703			
Personal Norms (PN)	PN1		0.671	0.839	0.839	0.567
	PN2	13.217***	0.786			
	PN3	12.880***	0.758			
	PN4	13.270***	0.791			
Cognitive Empowerment (CE)	CE1		0.719	0.789	0.794	0.563
	CE2	13.181***	0.782			
	CE3	12.866***	0.748			
Emotional Empowerment (EE)	EE1		0.788	0.826	0.828	0.616
	EE2	15.136***	0.782			
	EE3	15.166***	0.784			
Behavioral Empowerment (BE)	BE1		0.784	0.828	0.828	0.617
	BE2	16.413***	0.815			
	BE3	15.259***	0.756			
Autonomous Driving Technology Acceptance (AC)	AC1		0.727	0.805	0.806	0.582
	AC2	14.730***	0.831			
	AC3	13.323***	0.725			

**Table 5:** Discriminant validity.

	SN	PN	CE	EE	BE	AC
SN	0.748					
PN	0.378	0.753				
CE	0.606	0.345	0.750			
EE	0.615	0.399	0.455	0.785		
BE	0.667	0.527	0.528	0.553	0.785	
AC	0.627	0.538	0.583	0.542	0.715	0.763

Note: $\text{Diagonal}\;=\sqrt{AVE}$; off-diagonals = inter-construct correlations. SN for subjective norms, PN for personal norms, CE for cognitive empowerment, EE for emotional empowerment, BE for behavioral empowerment, and AC for acceptance of autonomous driving technology.

### 3.3. Results of hypothesis testing

**Fig 2:**
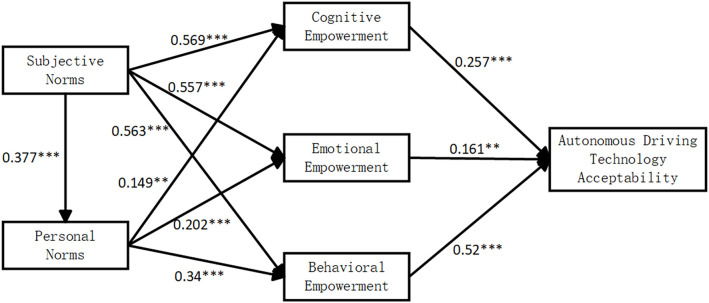
Results of model testing.

Using AMOS software, we tested the proposed hypotheses. The results, shown in Table 6, indicate that all three dimensions of psychological empowerment have a significant positive effect on the public acceptance of autonomous driving technology. Personal norms positively influence all three dimensions of psychological empowerment, while subjective norms significantly enhance both personal norms and the three dimensions of psychological empowerment, as depicted in Fig 2.

**Table 6:** Results of hypothesis testing.

Hypotheses	Paths	Path factor	Critical Ratio	Hypothetical results
H1	CE-- > AC	0.257***	4.326	supported
H2	EE-- > AC	0.161**	2.822	supported
H3	BE-- > AC	0.520***	7.682	supported
H4	PN-- > CE	0.149**	2.598	supported
H5	PN-- > EE	0.202***	3.638	supported
H6	PN-- > BE	0.340***	6.406	supported
H7	SN-- > CE	0.569***	8.446	supported
H8	SN-- > EE	0.557***	8.893	supported
H9	SN-- > BE	0.563***	9.451	supported
H10	SN-- > PN	0.377***	6.322	supported

Note:* denotes p < 0.05,** denotes p < 0.01,*** denotes p < 0.001, SN for subjective norms, PN for personal norms, CE for cognitive empowerment, EE for emotional empowerment, BE for behavioral empowerment, and AC for acceptance of autonomous driving technology.

### 3.4. Test of the mediating effect of user psychological empowerment

Using PROCESS in SPSS, we conducted a mediating effect analysis, as shown in Table 7. The results reveal that psychological empowerment partially mediates the relationship between subjective norms and the public acceptance of autonomous driving technology, as well as between personal norms and the public acceptance of autonomous driving technology.

**Table 7:** Results of the mediation effect.

Paths	c	a	b	a*b	a*b	c’	Mediation type
	Total effect			Indirect effect	(95%BOOTCI)	Direct effect	
SN-- > CE -- > AC	0.423***	0.470***	0.243***	0.114***	0.062-0.172	0.308***	Partial mediation effects
SN-- > EE -- > AC	0.423***	0.565***	0.176***	0.099***	0.055-0.148	0.323***	Partial mediation effects
SN-- > BE -- > AC	0.423***	0.575***	0.339***	0.195***	0.135-0.257	0.228***	Partial mediation effects
PN-- > CE -- > AC	0.335***	0.256***	0.322***	0.082***	0.043-0.131	0.253***	Partial mediation effects
PN-- > EE -- > AC	0.335***	0.342***	0.246***	0.084***	0.049-0.124	0.251***	Partial mediation effects
PN-- > BE -- > AC	0.335***	0.430 ***	0.386***	0.166***	0.117-0.220	0.169***	Partial mediation effects

Note:* denotes p < 0.05,** denotes p < 0.01,*** denotes p < 0.001, SN for subjective norms, PN for personal norms, CE for cognitive empowerment, EE for emotional empowerment, BE for behavioral empowerment, and AC for acceptance of autonomous driving technology.

### 3.5. Importance-performance analysis

This study employed Importance-Performance Analysis (IPA) to examine the differential impacts of psychological empowerment dimensions on the public acceptance of autonomous driving technology. Notably, the performance analysis evaluates how effectively these factors enhance user acceptance. Importance values were derived from the path coefficients in the structural equation model, while performance scores, ranging from 0 to 100, were based on latent variable scores. The calculation of these scores follows the consumer satisfaction index formula proposed by Anderson and Fornell (2000) [96], as detailed in Equation (1).


$$\begin{array}{c} \varepsilon_{\text{i}}=\frac{\text{E}\left[\varepsilon_{\text{i}}\right]-min\left[\varepsilon_{\text{i}}\right]}{max\left[\varepsilon_{\text{i}}\right]-min\left[\varepsilon_{\text{i}}\right]}*\text{100} \end{array}$$ (1)

In this context, the $\varepsilon_{\text{i}}$ values for each latent variable are based on the average values of their corresponding observed variables. The importance and performance values are presented in Table 8.

**Table 8:** Path coefficients and scores.

Latent variables	Scores	Acceptance of Autonomous Driving Technology		
		Path factors	Critical Ratios	P-values
AC	43.73	–	–	–
CE	33.90	0.257	4.326	<0.001
EE	47.28	0.161	2.822	<0.01
BE	51.19	0.520	7.682	<0.001

Note:* denotes p < 0.05,** denotes p < 0.01,*** denotes p < 0.001, SN for subjective norms, PN for personal norms, CE for cognitive empowerment, EE for emotional empowerment, BE for behavioral empowerment, and AC for acceptance of autonomous driving technology.

**Fig 3:**
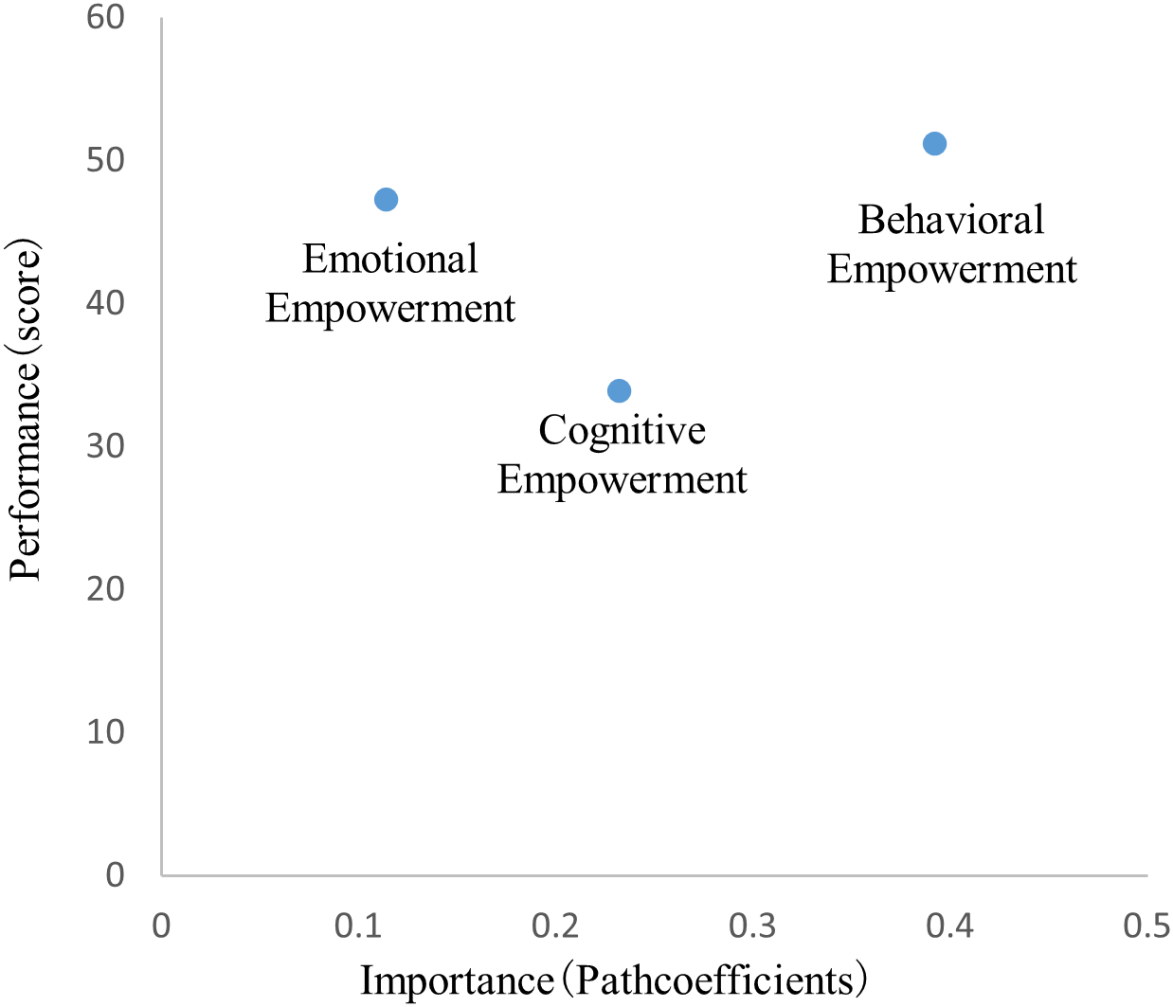
Importance-performance analysis.

The data analysis results, presented in Fig 3, indicate that, in comparison to emotional and cognitive empowerment, behavioral empowerment demonstrates higher importance and performance.

### 3.6 Analysis of Bayesian network models

**Fig 4:**
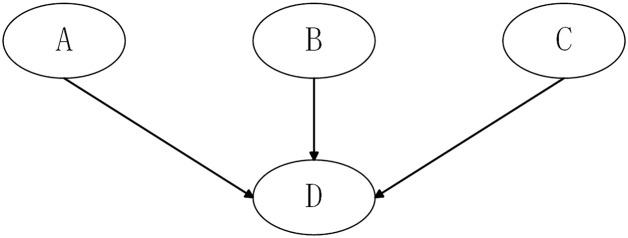
Schematic diagram of a Bayesian network model.


To validate the proposed model, a Bayesian network structure model was constructed to evaluate the results of the structural equation modeling. In Fig 4, each parent node represents the cause, while the child node signifies the outcome. Arrows indicate causal relationships, forming a directed acyclic graph (DAG). The parent set of a node D is denoted as parent(D), and the joint distribution of node values is expressed as the product of the local distributions of each node and its parent, as shown in Equation (2).

$$\begin{array}{c} 𝐏(A,B,C)=\prod p\left(D|parents(D)\right) \end{array}$$ (2)

In this study, the hypothesis testing from the SEM served as the foundational structure for the Bayesian network (BN). Prior to estimating the conditional probabilities, node discretization was performed. The discretized values for the nodes were obtained through factor scoring from the SEM, which served as the raw data for BN modeling. Given the characteristics of the data, the observed variables for subjective norms and personal norms each comprised four items, while the remaining latent variables each had three observed variables, all measured using a 5-point Likert scale. For subjective norms and personal norms, the latent variable scores were discretized into “low” (scores ≥1 and ≤2.5), “medium” (scores >2.5 and <3.5), and “high” (scores ≥3.5 and ≤5) states. For cognitive empowerment, emotional empowerment, behavioral empowerment, and the public acceptance of autonomous driving technology, the latent variable scores were discretized into “low” (scores ≥1 and ≤2.33), “medium” (scores >2.33 and <3.67), and “high” (scores ≥3.67 and ≤5) states. The node state divisions are shown in Table 9.

**Table 9:** Division table of node states.

	SN	PN	CE	EE	BE	AC
Low	[1–2.5]	[1–2.5]	[1–2.33]	[1–2.33]	[1–2.33]	[1–2.33]
Medium	(2.5–3.5)	(2.5–3.5)	(2.33–3.67)	(2.33–3.67)	(2.33–3.67)	(2.33–3.67)
High	[3.5–5]	[3.5–5]	[3.67–5]	[3.67–5]	[3.67–5]	[3.67–5]

**Fig 5:**
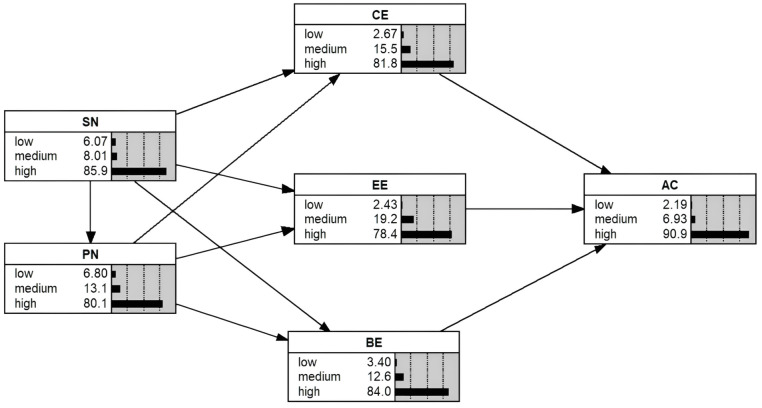
Bayesian network after updating using EM algorithm.

Table 10 presents the prior probability distributions for each node state in the questionnaire. Specifically, for the “public acceptance of autonomous driving technology” node, 1.94% of participants exhibited “low” acceptance, 8.25% demonstrated “medium” acceptance, and 89.81% showed “high” acceptance. Due to the inclusion of unobserved latent variables in the network structure, missing data were present in the BN system. However, the Expectation-Maximization (EM) algorithm can address such missing data and automatically compute the Conditional Probability Tables (CPT) in the BN. Consequently, the EM algorithm in Netica software was applied to develop and update the BN model, as shown in Fig 5.

**Table 10:** Distribution table of prior probabilities of states of each node.

State	variant					
	SN	PN	CE	EE	BE	AC
Low	6.07%	6.80%	2.67%	2.43%	3.40%	1.94%
Medium	8.01%	13.11%	15.53%	19.17%	12.62%	8.25%
High	85.92%	80.10%	81.80%	78.40%	83.98%	89.81%

After determining the structure of the Bayesian Network (BN) model, the original dataset was used to evaluate its accuracy. The error rate was chosen as the evaluation metric. For model training, 70% of the original dataset was randomly selected as the training set, while the remaining 30% was used as the test set. The validation results are summarized in Table 11. The BN model demonstrated an error rate of 0% for both “low” and “high” acceptance levels, and an error rate of 11.11% for “medium” acceptance. The overall error rate was 2.44%. These findings suggest that the proposed model offers strong predictive capability for forecasting public acceptance of autonomous driving technology.

**Table 11:** Bayesian network performance evaluation results.

Confusion matrix				Error rate	Total error rate
Projected value			Actual value		
Low	Medium	High			
2	0	0	Low	0.00%	2.44%
0	16	2	Medium	11.11%	
0	0	62	High	0.00%	

## 4. Discussion

### 4.1. General discussion

This study adopted behavioral intention (BI) as the primary outcome rather than observed behavior. The approach is standard in technology-acceptance research and is supported by evidence that BI predicts subsequent use, including for emerging technologies such as autonomous driving. Although intention–behavior gaps can arise, BI is a reliable proxy in early-adoption contexts and offers actionable insights for research and practice [9,97]. To enhance external validity, future work should pair BI with longitudinal follow-ups or real-world trials to test whether intentions translate into actual use.

All hypothesized paths were supported. Subjective norms and personal norms each showed significant positive effects on acceptance, and all three dimensions of psychological empowerment—cognitive, emotional, and behavioral—were significant and positive. Subjective norms also strengthened personal norms, consistent with prior evidence on normative influences in technology adoption [18,24,27,32,56–59,61–63]. In China’s context of Confucian heritage, collectivism, and distinctive institutions, such normative pathways may be amplified, whereas patterns may differ in more individualistic settings. Path comparisons indicated that subjective norms exceeded personal norms on all three empowerment dimensions. All coefficients were positive and significant, implying that external social and institutional signals translated into perceived capability and readiness more efficiently than internally anchored moral commitments. Personal norms remained meaningful: many respondents appeared to view support for autonomous driving as value-consistent, plausibly reflecting the education profile (≈95% with at least high-school education), which correlates with exposure and willingness to adopt, consistent with Liljamo [54]. H10 was supported: subjective norms reinforced personal norms, aligning with evidence that moral intuitions often track prevailing subjective norms [33,60,79,98]. Mediation analyses placed psychological empowerment at the center: it partially transmitted both normative pathways to acceptance, with behavioral empowerment providing the strongest conduit and the largest direct effect. This dominance likely reflects proximity to the acceptance indicator (behavioral intention) and the tendency for action to follow understanding and positive affect, whereas cognition or emotion alone may not culminate in behavior [37,61]. Users therefore prioritized tangible, behavior-level benefits; design and communication should activate all three empowerment dimensions—capability and controllability (behavioral), transparency and risk–benefit clarity (cognitive), and positive affect (emotional)—to promote durable adoption.

Methodologically, the Bayesian network (BN) complemented the SEM results. The BN used prior and sample-space probabilities to estimate posterior distributions and updated variable probabilities in light of new observations. Unlike SEM, the BN offered probabilistic model-fit and parameter-estimation tools and graphically represented relationships among variables; here it was optimized with the Expectation–Maximization algorithm, helping ensure data completeness and improving the precision of predictions for autonomous-driving acceptance.

Finally, cultural and urban context shaped how the model operated. The empowerment-based framework worked well in China, yet applicability may vary elsewhere. Chinese Confucian culture emphasizes collectivism and societal expectations, which made subjective norms more influential; decisions were shaped by personal judgment and by social and governmental guidance. By contrast, the TAM lineage originated in Western individualistic contexts and may not fully capture the salience of social responsibility and moral obligation in collectivist cultures; applicability in China therefore required cultural adjustment [99]. Personal norms were particularly salient in China, where behavior is often driven by responsibilities to family and society. Cross-cultural comparisons should examine the joint effects of social norms, personal norms, and empowerment to test universality and scope conditions [100]. Within China, cities were heterogeneous: Tier-1 pilot hubs with richer media ecosystems and frequent AV trials may have converted social influence into intention more efficiently than lower-tier or inland cities, where risk–benefit clarity and trust in local governance could weigh more on cognitive and emotional empowerment. Migration patterns, hukou-based service access, and digital infrastructure further shaped exposure to AVs and the speed of norm diffusion, helping explain why the same intervention yielded different acceptance profiles across urban China.

### 4.2. Implications

Importance–Performance Analysis indicates that behavioral empowerment holds both the highest importance and the highest performance for autonomous-driving acceptance; it should be sustained though headroom for further gains is limited. Emotional empowerment shows high performance but lower importance, warranting maintenance rather than major investment. Cognitive empowerment has the lowest performance with moderate influence, providing the primary target for enhancement.

These patterns translate into three application domains. Market strategy should foreground tangible, behavior-level benefits that heighten felt capability and controllability, reduce effort, and build confidence; messaging can leverage social and institutional signals to convert norms into empowerment and foster adoption. Technology development can use the model as a tool to prioritize features that strengthen behavioral empowerment while systematically raising cognitive empowerment through transparent functions, clear risk–benefit explanations, and understandable performance feedback, thereby aligning product roadmaps with likely adoption levers. Intelligent ecosystem planning should integrate social-psychological factors into AV deployment—designing communication, governance, and service environments that respect cultural context—so that diffusion is both sustainable and socially aligned.

### 4.3. Limitations and future research

(1) Limitations in data collection. Data were collected via an online questionnaire. Although respondents were briefed on autonomous driving, many lacked direct experience, so some answers reflected hypothetical scenarios and potential bias. Future work should conduct external validity checks against independent datasets to strengthen reliability.
(2) Intent–behavior discrepancy. Acceptance was measured as behavioral intent, which often diverges from actual use. Because regional deployment of autonomous driving is uneven, observing real usage for all participants was infeasible. Longitudinal tracking and revealed-preference data are needed to improve accuracy and relevance.
(3) Model scope beyond UTAUT. Our model is partly constrained by the UTAUT paradigm. As AI ethics gains prominence, alternative theories may better capture engagement and risk perception. For example, Trinh et al. [101] highlight socio-cultural drivers of acceptance, and How Humans Judge Machines shows that cultural background and ethical responsibility shape judgments of AI [102]. Future research should integrate social-cognition and ethical frameworks to provide a more comprehensive account of AI risk and social acceptance.
(4) Validating Urban–Cultural Moderation. Future work will use multilevel, multi-city designs with objective and perceived exposure proxies, brief framing experiments, and links from intentions to pilot usage records.

## 5. Conclusion

This study integrates subjective norms and personal norms with the three facets of psychological empowerment to explain Chinese public acceptance of autonomous driving. Using CB-SEM on a sample of 412 respondents, we find that behavioral empowerment exerts the strongest direct effect on acceptance (β ≈ 0.52), exceeding cognitive (β ≈ 0.26) and emotional empowerment (β ≈ 0.16). All three empowerment dimensions partially mediate the effects of subjective and personal norms, with behavioral mediation dominating (≈46–50%). Importance–Performance Analysis locates behavioral empowerment in the high-importance/high-performance quadrant, while cognitive empowerment shows the greatest headroom for improvement. A Bayesian network trained on the SEM structure confirms predictive validity with a low overall error rate (≈2.44%).

Theoretically, these results extend acceptance research by positioning empowerment—not only attitudes or utility—as the proximal pathway translating social norms into adoption intent within a collectivist, top-down cultural context. Practically, they suggest that design and policy should prioritize features and services that amplify users’ felt capability and controllability (e.g., clear takeover protocols, reliable handover feedback, situational explanations, and demonstrable safety benefits), while using institutional signaling and social proof to activate norms. Given the performance–importance profile, investments that lift cognitive empowerment—such as transparency, tutorial/on-road demonstrations, and risk-benefit communication—are likely to yield incremental gains beyond already strong behavioral empowerment; emotional experiences remain supportive but secondary.

## Supporting information

S1 File Raw data. The minimal data set.
(XLSX)
